# External validation of the Tyrolean hip arthroplasty registry

**DOI:** 10.1186/s40634-022-00526-3

**Published:** 2022-08-30

**Authors:** Moritz Wagner, Sabrina Neururer, Dietmar Dammerer, Paul Nardelli, Gerhard Kaufmann, Matthias Braito, Alexander Brunner

**Affiliations:** 1 Department of Orthopaedic Surgery, District Hospital St. Johann, Bahnhofstraße 14, 6380 St. Johann, Austria; 2 PMU, Paracelsus Medical University, Salzburg, Austria; 3 Institute for Clinical Epidemiology, Tirol Kliniken, Anichstraße 35, 6020 Innsbruck, Austria; 4 Department for Orthopaedics and Traumatology, University Hospital Krems, Krems an der Donau, Austria; 5 Department of Orthopaedics and Traumatology, Medical University of Innsbruck, Anichstrasse 35, 6020 Innsbruck, Austria; 6 OFZ Innsbruck, Innrain 2 / 3. Stock, 6020 Innsbruck, Austria

**Keywords:** Adherence, Coverage, Revision, MDR, Regional, National

## Abstract

**Purpose:**

Arthroplasty registries gained increasing importance to the re-certification of orthopaedic implants according to the European Union (EU) Medical Device Regulation (MDR) adopted in 2017. Until recently, several European countries only had regional arthroplasty registries. Whether regional registries deliver data quality comparable with national registries remained unclear. Therefore, the purpose of this study was to validate the Austrian Tyrolean Hip Arthroplasty Registry (THAR) and to evaluate if this regional registry showed adequate adherence, completeness and correctness when compared with well-established national registries.

**Methods:**

A consecutive series of 1100 primary total hip arthroplasties were identified from our institution’s medical database. Patients were interviewed by phone and completed questionnaires after a mean follow-up period of 8.05 years and were asked if they had had revision surgeries. The data were compared to the corresponding dataset from the THAR.

**Results:**

Adherence was 97.91% for primary total hip arthroplasty. Clinical follow-up identified 10 missing cases, resulting in adherence of 81.48% for revisions. Completeness of patient-reported outcome measurements was 78.55% before surgery and 84.45% 1 year after surgery. Correctness was 99.7% for demographic data, 99.54% for implant specifications, and 99.35% for mode of fixation.

**Conclusion:**

The data of this study showed that regional arthroplasty registries can deliver data quality comparable with well-established national registries. The main reason for unrecorded revision cases and wrongly recoded implants was human error. Further digitalization with more automatic data submission may have the potential to reduce these failure rates in the future. Overall, the THAR represents a valid data source for re-certification of medical implants according to the EU’s MDR.

## Introduction

The last revision of the Medical Device Regulation (MDR), adopted by the European Union (EU) parliament in April 2017, was intended to improve patient safety by enhancing transparency and traceability of medical products [5, 16]. In addition, it required thorough device assessment prior to and post release on the EU single market [16]. As a result, orthopaedic implants that have been established for many years will have to be re-certified based on clinical data until 25 May 2025. Considering that around 25% of all total hip replacement implants available in the United Kingdom still have no scientific evidence for their clinical effectiveness, re-certification may represent a major challenge for many manufacturers [8, 9]. Therefore, the data available from joint arthroplasty registries are of increasing interest, not only to scientists but also to medical companies. In Europe, 15 national hip arthroplasty registries exist, recording around 3.1 million primary total hip arthroplasty (THA) procedures. In several countries, including Austria, Italy and Spain, only regional state registries exist [10]. A recent evaluation of England’s National Joint Registry (NJR) has shown that around 24% of all revision surgeries are recorded incorrectly [1]. This rate may be even higher for regional state registries as they usually do not cover revision events performed across state lines [11].

In Austria, the Tyrolean Hip Arthroplasty Registry (THAR) is the only well-established joint registry in the country. It is a regional state registry containing data from 10 hospitals that conduct joint replacement surgeries in the state of Tyrol. Until 2025, a number of total hip implants will have to be re-certified on the basis of data provided by the THAR [17, 18]. Therefore, the purpose of this study was to validate the THAR and to assess whether a regional registry can deliver data quality comparable with national registries. We hypothesized that the evaluated regional registry may underreport revision cases compared with national registries.

## Patients and methods

This clinical study was approved by the ethical committee of the Medical University Innsbruck (1224/2020) and was conducted in accordance with the Declaration of Helsinki.

### The tyrolean hip arthroplasty registry

**Fig. 1:**
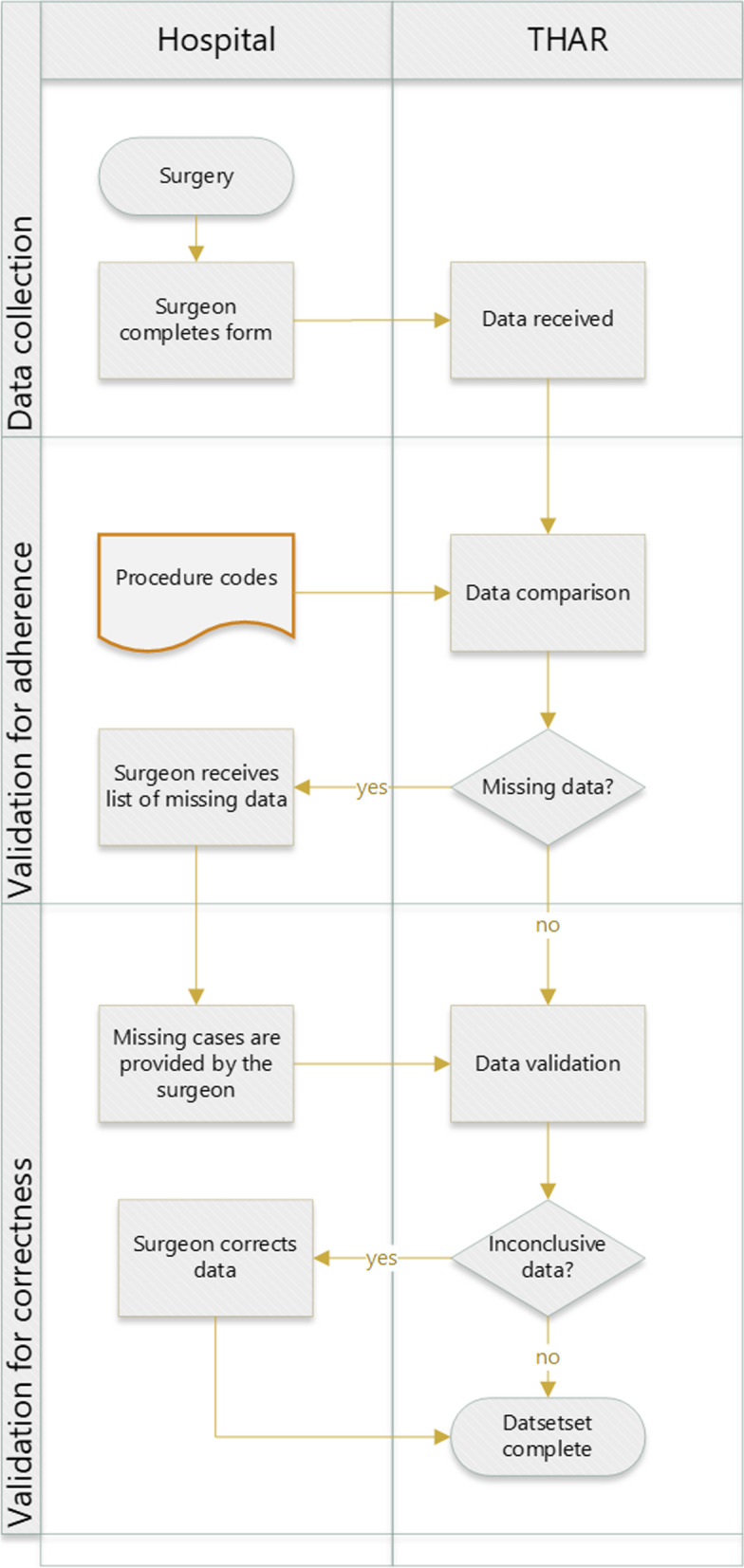
Flowchart of the internal validation process for data from the THAR. THA: Total hip arthroplasty

The THAR is a regional arthroplasty registry of the Department of Clinical Epidemiology at the Tyrolean Federal Institute for Integrated Care (Tirol Kliniken GmbH, Innsbruck, Austria). Data from 10 hospitals that perform joint replacement surgeries within an area of 4880 square miles (12,650 km^2^) and covering a population of 757,634 (as of 31 December 2019) were recorded on the registry. The records start from the year 2004 and contain data on primary and revision arthroplasties of the hip. Demographic data, indications for surgery, name and type of implant, mode of fixation, and surgical approach are collected from the surgeon. Patients complete the self-administered Western Ontario and McMaster Universities Osteoarthritis Index (WOMAC) questionnaire 1 day before and 1 year after arthroplasty [13]. This registry annually covered around 2850 hip arthroplasties (primary and revision), and it was estimated that 97.5% of all primary arthroplasties performed on citizens of the region are covered. A recent estimation indicated that 171–210 elective hip arthroplasties are conducted per 100,000 inhabitants. The overall revision rate within 4 years after primary hip arthroplasty was reported as 4.12%. The self-reported level of adherence is 99.2%. The THAR deploys an internal validation process to ensure adherence and correctness are high, and data from medical insurers and the national death registry, as well as checks for plausibility, are embedded (Fig. 1).

### Validation of the THAR

This study evaluated two consecutive series of patients who received primary total hip arthroplasty at our institution (BKH St. Johann, Tyrol, Austria) in the periods 2008–2012 and 2014–2016. Our institution is one of 10 hospitals that are covered by the THAR. It is located in proximity to the geographic state border. Only patients treated with a Siocon pressfit cup and a Monocon [18] or MonoconMIS [17] stem (Falcon Medical, Mödling, Austria) were included. Overall, a total of 1013 patients (corresponding to 1100 primary arthroplasties) met the inclusion criteria. The hospital records of those primary arthroplasties were gathered in a retrospective manner compared to the THAR.

The second part of this study included a prospective clinical follow-up of all included patients, with the aim to identify revision arthroplasties. According to their social security records, 125 patients had died following index arthroplasty. The medical files of the remaining 888 patients were obtained from our institution’s database. Patients were contacted by mail and phone and asked if they had undergone revision surgery. If patients reported revision surgeries that had been performed at external institutions, we requested medical records from the corresponding hospitals. A total number of 606 patients could be followed after a mean period of 8.05 years (standard deviation: 3.2). A total of 238 patients were lost to follow-up (response rate = 71.8%). Those revision arthroplasties were compared to the THAR.

Only the surgical report was considered proof of an arthroplasty. Adherence, completeness and correctness of the THAR were thereby assessed, both, for retrospectively identified primary arthroplasties and for prospectively confirmed revision arthroplasties [3].

### Statistical analysis

Descriptive statistics were used to describe the dataset. Categorical values were presented as absolute and relative frequencies. Numerical variables were expressed as means, differences between groups have been compared with the student’s paired t-test. The level of significance was set at ≤ 0.05. In this study, the number of primary and revision joint arthroplasties was evaluated. The statistical evaluation has been performed using IBM SPSS Statistics version 27 (IBM, New York, USA).

## Results

### Adherence for primary total hip arthroplasty

A total of 1100 primary arthroplasties were documented in our institutional medical records, of which 1077 were recorded by the THAR, resulting in an adherence of 97.91% for primary THA.

### Adherence for revision total hip arthroplasty

**Fig. 2:**
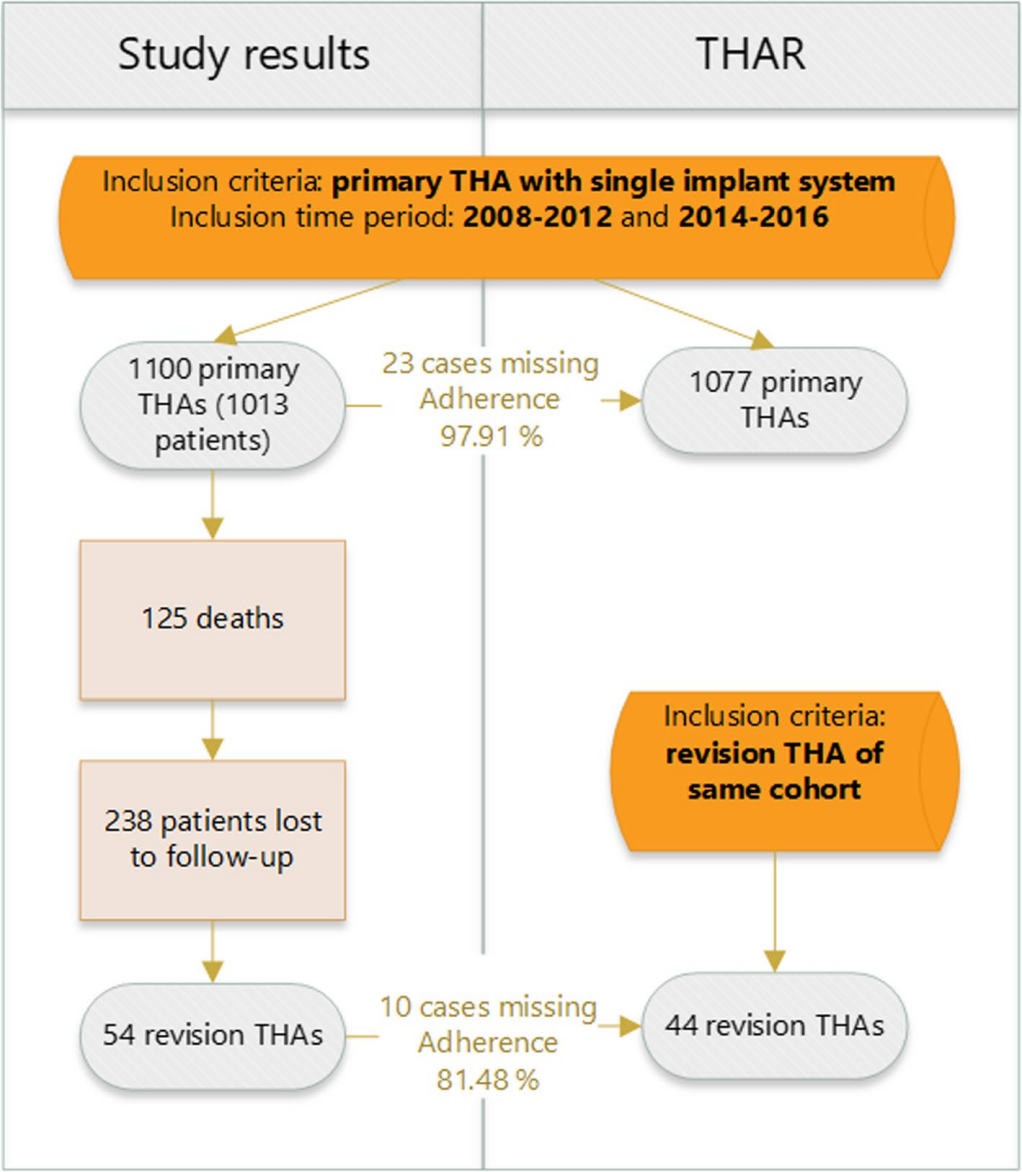
Flow diagram for prospective clinical follow-up of revision surgeries. THA: Total hip arthroplasty

A total of 44 revision arthroplasties were identified from the THAR. Fifty-four revisions were identified by clinical follow-up, resulting in an adherence of 81.48% for revision arthroplasty (Fig. 2). The reasons for the 10 missing revision cases were investigated. In four cases, the revision surgery was performed at a hospital outside the area covered by the THAR. In three cases, the surgeon failed to report the revision surgery and the wrong surgery procedure code was recorded (two in our institution and one at an external hospital covered by the THAR). In two cases, the revision arthroplasty was performed in our institution within 2 days after the index procedure and was erroneously not classified as revision. In one case, the revision arthroplasty was not yet included in the registry’s dataset and was still in the internal validation process.

### Completeness

Perioperative data, including demographics, clinical information, intraoperative details, and implant specifications, were complete. The completeness of patient-reported outcome measurement (WOMAC) before the primary arthroplasty was 78.55% (*n* = 864) and was 84.45% (*n* = 929) 1 year after surgery. Patients who completed the patient-reported outcome measurements were significantly younger (65.7 years versus 69.4 years; *p* < 0.001) and were more frequently male (437 out of 741 males versus 427 out of 876 females; *p* < 0.001).

### Correctness for primary total hip arthroplasty

Demographic data, including name, date of birth and reported sex, were correct in 1067 (99.07%) cases. Clinical information was correct in all cases. Implant specifications were correct in 1072 (99.54%) cases. Mode of fixation (cementless versus cemented) was correct in 1070 (99.35%) cases. In three patients, the wrong site was captured by the arthroplasty registry (level of correctness: 99.72%).

### Correctness for revision total hip arthroplasty

Demographic data and clinical data were correct in all of 44 recorded revision cases. The wrong implant was recorded in 3 out of 44 cases (correctness: 93.18% for implants) and the wrong mode of fixation was recorded in 2 out of 44 cases (correctness: 95.45% for the fixation mode). Study results are summarized in Table 1.

**Table 1:** Comparison of previous estimates and reported levels of adherence, completeness, and correctness of the THAR with national joint replacement registries. THA: Total hip arthroplasty; WOMAC: Western Ontario and McMaster Universities Osteoarthritis Index

Source	Data	Adherence	Completeness	Correctness
THAR	Primary THA	97.91% (*n* = 1100)	78.55%	99.54%
	Revision THA	81.48% (*n* = 54)	n/a	93.18%
New Zealand Joint Registry [12]	Primary THA	96.3% (*n* = 28,970)	n/a	n/a
	Revision THA	92.5% (*n* = 4263)	n/a	n/a
UK National Joint Registry [1]	Revision THA	79.6% (*n* = 9411)	n/a	n/a
Norwegian Arthroplasty Register [2]	Primary THA	99.6% (*n* = 85,082)	n/a	n/a
	Revision THA	97.8% (*n* = 14,359)	n/a	n/a
Finnish Arthroplasty Registry [15]	Primary THA	96.1% (*n* = 2820)	n/a	n/a

## Discussion

In this study, the THAR showed comparable adherence, completeness and correctness with information reported on national registries [1, 2, 7, 15]. Likewise, the lower adherence for revision surgeries was in accordance with validation studies for national registries [2, 4, 15] (Table 1).

Although several European countries had only regional state arthroplasty registries until recently, validation studies on regional registries remain sparse in the literature. Stea et al. (2009) reported an adherence of 93% for primary total hip arthroplasty for the regional registry of the Emilia-Romagna region in Italy [14]. Bautista et al. reported a level of adherence of 98.6% for primary arthroplasties for the institutional registry at the University Hospital in Santa Fe de Bogota on a random sample of 53 patients [3]. None of those studies reported on adherence for revision surgeries.

As stated by Pugley et al. (7), regional registries have an increased potential for under-recording revision surgeries as some procedures may be performed at hospitals not covered by the registry [11]. Our institution is located in proximity to the district’s border. In our study, only 4 of the 54 revisions were performed at hospitals not covered by the THAR. These data suggest that the limited geographic coverage of the THAR has little impact on adherence for revisions.

The most frequent mode of failure was the human error of the surgeon not reporting the procedure as a revision. Overall, around 10% of revision procedures were not recorded, as the surgeon entered the wrong procedure code. In comparison, a recent evaluation of England’s NJR revealed mal-coding rates for revisions of around 15% [7]. Even higher rates were reported for France’s joint registry [6].

Completeness and correctness of demographic data were very high in our study, as these parameters are automatically digitally submitted from our medical data software to the THAR. Failures in recording the correct implant, the mode of fixation or the side of the operated hip were again predominately a matter of human error.

One major limitation of this study is that we included only patients from one institution. However, since the modes of data acquisition and data transfer were similar in all hospitals covered by the THAR, this selection bias may be of minor impact.

In addition, we assessed the rate of revision events based on patient self-reporting. Prior evaluations have shown that self-reporting of arthroplasty has a sensitivity of around 95% [15]. Therefore, the actual revision rate of our patients may collectively be slightly higher than reported. Strengths of this study were its large sample, and the relatively long follow-up period after which patients were contacted personally. To our knowledge, this was the first external validation study of the THAR and one of very few studies with clinical follow-up to identify revision surgeries.

## Conclusion

The data of this study showed that regional arthroplasty registries can deliver data quality comparable with established national registries. The main reason for unrecorded revision cases and wrongly recorded implants was human error. Further digitalization with more automatic data submission may have the potential to reduce these failure rates in the future. Overall, the THAR represented a valid data source for re-certification of medical implants according to the EU’s MDR.

## Data Availability

All data generated or analysed during this study are included in this published article. This study or contents of this study have not been published or submitted for publication elsewhere.
